# Retraction Note: The hematological impact of umbilical cord milking versus delayed cord clamping in premature neonates: a randomized controlled trial

**DOI:** 10.1186/s12884-026-09326-4

**Published:** 2026-05-26

**Authors:** Hytham Atia, Ahmed Badawie, Osama Elsaid, Mahmoud Kashef, Nourhan Alhaddad, Mohamed Gomaa

**Affiliations:** 1 Faculty of Medicine, Zagazig University, Zagazig, Sharkia Egypt; 2 Armed Forces Hospitals Southern Region, King Faisal Military City Hospital, Base-villa 68, King Faisal military city, 62417-8183, Khamis Mushait, Kingdom of Saudi Arabia; 3 Faculty of Medicine, October 6 University, Cairo, Egypt; 4 Al Ahmady Hospital, Madina, Kingdom of Saudi Arabia

**Retraction Note: BMC Pregnancy and Childbirth 22, 714 (2022)**

**https://doi.org/10.1186/s12884-022-05046-7**

This article has been retracted by the Editor. Following publication, concerns were raised regarding the randomisation process, in that several of the baseline characteristics reported in Table 1 are significantly different between the two groups, which would not be expected to have arisen as a result of adequate randomisation process. Post publication statistical review confirmed the concerns raised regarding the randomisation process and concluded that this may have impacted the results and conclusions. The Editor therefore no longer has confidence in the results and conclusions of this article. The authors have been offered the opportunity to submit a revised version of their article to the journal addressing all the concerns raised, which would undergo full peer review.

Authors Hytham Atia, Ahmed Badawie, Osama Elsaid, Mahmoud Kashef, Nourhan Alhaddad and Mohamed Gomaa did not respond to correspondence from the Publisher about this retraction.

